# Association of Extensive Video Gaming and Cognitive Function Changes in Brain-Imaging Studies of Pro Gamers and Individuals With Gaming Disorder: Systematic Literature Review

**DOI:** 10.2196/25793

**Published:** 2021-07-09

**Authors:** Eunhye Choi, Suk-Ho Shin, Jeh-Kwang Ryu, Kyu-In Jung, Yerin Hyun, Jiyea Kim, Min-Hyeon Park

**Affiliations:** 1 Department of Psychiatry Eunpyeong St. Mary’s Hospital, College of Medicine The Catholic University of Korea Seoul Republic of Korea; 2 Dr. Shin’s Child and Adolescent Psychiatry Clinic Seoul Republic of Korea; 3 Department of Physical Education College of Education Dongguk University Seoul Republic of Korea

**Keywords:** brain imaging, cognitive function, gaming disorder, pro gamers, video games, cognition, brain, games, gaming

## Abstract

**Background:**

The World Health Organization announced the inclusion of gaming disorder (GD) in the International Classification of Diseases, 11th Revision, despite some concerns. However, video gaming has been associated with the enhancement of cognitive function. Moreover, despite comparable extensive video gaming, pro gamers have not shown any of the negative symptoms that individuals with GD have reported. It is important to understand the association between extensive video gaming and alterations in brain regions more objectively.

**Objective:**

This study aimed to systematically explore the association between extensive video gaming and changes in cognitive function by focusing on pro gamers and individuals with GD.

**Methods:**

Studies about pro gamers and individuals with GD were searched for in the PubMed and Web of Science databases using relevant search terms, for example, “pro-gamers” and “(Internet) gaming disorder.” While studies for pro gamers were searched for without date restrictions, only studies published since 2013 about individuals with GD were included in search results. Article selection was conducted by following the PRISMA (Preferred Reporting Items for Systematic Reviews and Meta-Analyses) guidelines.

**Results:**

By following the PRISMA guidelines, 1903 records with unique titles were identified. Through the screening process of titles and abstracts, 86 full-text articles were accessed to determine their eligibility. A total of 18 studies were included in this systematic review. Among the included 18 studies, six studies included pro gamers as participants, one study included both pro gamers and individuals with GD, and 11 studies included individuals with GD. Pro gamers showed structural and functional alterations in brain regions (eg, the left cingulate cortex, the insula subregions, and the prefrontal regions). Cognitive function (eg, attention and sensorimotor function) and cognitive control improved in pro gamers. Individuals with GD showed structural and functional alterations in brain regions (eg, the striatum, the orbitofrontal cortex, and the amygdala) that were associated with impaired cognitive control and higher levels of craving video game playing. They also showed increased cortical thickness in the middle temporal cortex, which indicated the acquisition of better skills. Moreover, it was suggested that various factors (eg, gaming expertise, duration or severity of GD, and level of self-control) seemed to modulate the association of extensive video game playing with changes in cognitive function.

**Conclusions:**

Although a limited number of studies were identified that included pro gamers and/or individuals who reported showing symptoms of GD for more than 1 year, this review contributed to the objective understanding of the association between extensive video game playing and changes in cognitive function. Conducting studies with a longitudinal design or with various comparison groups in the future would be helpful in deepening the understanding of this association.

## Introduction

### Background

Video game playing has become one of the most popular leisure activities [1]. With the growing popularity of video game playing, a minority of individuals have been reported to play video games in problematic ways, resulting in negative consequences (eg, withdrawal from socializing and death) [2-4]. By focusing on problematic video gaming in a minority of individuals, the World Health Organization (WHO) recently announced that the International Classification of Diseases, 11th Revision (ICD-11) included *gaming disorder* (GD) as a syndrome [5,6]. GD refers to the persistent engagement on the internet in playing games despite the psychological distress and the interference with daily activities for more than 12 months [7]. However, there are concerns about the inclusion of GD as one of diseases in the ICD-11 [6,8], in that the objective evidence that showed there were harmful effects of GD was not sufficient (ie, little research examined causality and the persistence of symptoms) [9].

After the announcement by the WHO, GD was reported in the media to result in structural alterations in brain regions based on the results of a cross-sectional study that compared the brain structures of individuals with GD to those of healthy controls; this emphasized the necessity of treatment for GD [10]. Although the tendency of GD was found to be negatively associated with the volume of gray matter (GM) in the prefrontal brain regions that are involved in cognitive control and sensorimotor functioning [11], it is difficult to confirm the causality of the association in the cross-sectional studies. Moreover, unlike the focus on GD, playing video games was positively associated with cognitive function [12-15]. Video game players, compared to non–video game players, showed more integrated white matter (WM) in motor and visual pathways [14] and higher levels of activation in the frontoparietal brain regions to detect visual stimulus despite the comparable cognitive performance [15]. Playing video games for a longer duration was also associated with thicker cortices in the brain regions for attention, navigation, visuomotor function, and the resolution of ambiguity (eg, the left frontal eye field, the left dorsolateral prefrontal cortex [DLPFC], and the bilateral entorhinal cortex) [12,13]. That is, while the association of GD with alterations in brain regions was more focused, playing video games was also associated with cognitive enhancement.

Furthermore, there are individuals who play video games extensively for more than 10 hours a day without reporting disrupted lifestyles (eg, a disrupted sleep-wake cycle) [16]; these individuals are called pro gamers. They refer to a group of people who belong to a team through a contract and who make economic profits by taking part in e-sports competitions [17]. The mean age of pro gamers in major leagues was reported to be 22 years [18]. Although statistics for their mean age of retirement were not available, more than half of pro gamers reported that their retirement was dependent on their judgment of their performances in competitions [17]. Since cognitive-motor speed (ie, the speed at which the cognitive process initiates actions) was found to start to decline at 24 years in a sample of StarCraft II players who were aged between 16 and 44 years old, regardless of their expertise level [19], pro gamers were assumed to retire at 25 to 27 years of age.

Taken together, playing video games for a longer duration did not result in the development of GD, and only a minority of people reported the development of GD [6,7]. Unlike negative opinions in the media toward video games and playing video games, playing them was associated with cognitive enhancement (eg, Zhang et al [14] and Richlan et al [15]). Video games were also suggested as a potential tool for clinical intervention for individuals with mental disorders (eg, Alzheimer disease) [20]. Thus, it is necessary to explore the association between video game playing and alterations in brain regions in a more objective manner. Moreover, despite the comparable amount of video game experience between individuals with GD and pro gamers who play video games extensively without any symptoms of GD (eg, higher impulsivity for gaming) [16], more studies have been conducted that focused on individuals with GD, and more review studies about GD have been conducted (eg, Leeman and Potenza [21] and Wei et al [22]). Since pro gamers, in addition to individuals with GD, are a population of interest for investigating the association between extensive video gaming and changes in cognitive function, reviewing studies that recruited pro gamers would deepen the understanding and effects of playing video games extensively.

### Objective

This systematic review aimed to explore the association between extensive video game playing and changes in cognitive function. That is, this study reviewed brain-imaging studies that included pro gamers and/or individuals with GD.

## Methods

### Search Strategy

Literature searches were conducted in two databases: PubMed and Web of Science. Studies about pro gamers were searched for with the following search terms, without a restriction on the date: “pro-gamers,” “pro video game players,” “action video game experts,” “video gaming experts,” and “long-term video game players.” Studies about individuals with GD were searched for with the following search terms, with date restrictions: “(Internet) gaming disorder,” “(Internet) gaming addiction,” and “(online) video game addiction.” As many studies about GD have been conducted, only studies that were published since 2013 were included in the results of the literature search.

### Study Selection

#### Overview

The PRISMA (Preferred Reporting Items for Systematic Reviews and Meta-Analyses) guidelines [23] were followed in this study. Firstly, duplicates from the search results from the two databases were removed using EndNote X9 (Clarivate Analytics). After the removal of duplicates, the titles and abstracts of the remaining articles were screened to determine if they were eligible for full-text assessment. Secondly, full-text articles were carefully reviewed to determine their eligibility for this review based on selection criteria.

#### Inclusion Criteria

Inclusion criteria for articles in this review were as follows: (1) original research articles published in English, (2) the use of brain-imaging techniques, and (3) the recruitment of pro gamers and/or individuals with GD. Definitions of pro gamers and GD in this study are as follows:

- Pro gamers are defined as individuals who (1) belong to e-sports teams, (2) are highly experienced video game players without reporting any problematic daily lifestyle behaviors, and/or (3) have won video game playing competitions.
- GD refers to persistent engagement in video game playing despite psychological distress and interference with daily activities [24] for more than 1 year [7].

That is, articles about pro gamers were selected for inclusion when recruited participants met at least one description of pro gamers above. For example, a study that recruited video game experts, who were recognized as top-ranking players [25], was included because the second description of pro gamers was met. The types of video games (eg, StarCraft and League of Legends) were not restricted for the selection. Articles were also selected for the review when recruited participants were confirmed to be diagnosed with GD for more than 12 months or when the stated mean duration of GD symptoms in participants was more than 1 year. In the process of the selection of articles about individuals with GD, the age of the individuals with GD was not restricted. This was because adults, in addition to adolescents, have also shown GD symptoms, despite the report that the prevalence of GD has increased especially in adolescents [26] whose cognitive control is developing [27] with different developmental trajectories of the limbic system and prefrontal cortex (PFC) regions [28].

#### Exclusion Criteria

Articles were excluded when full texts were not available and when participants in studies did not meet any of the descriptions of pro gamers defined above. Articles were also excluded when they did not confirm that individuals with GD showed GD symptoms for more than 1 year or when they did not present the information of the mean duration of GD.

### Data Extraction

The following data were extracted from the selected articles: information about the study (ie, study design, participants, duration of GD, and brain-imaging techniques used) and the brain regions that were associated with extensive video game playing.

## Results

### Literature Overview

**Figure 1:**
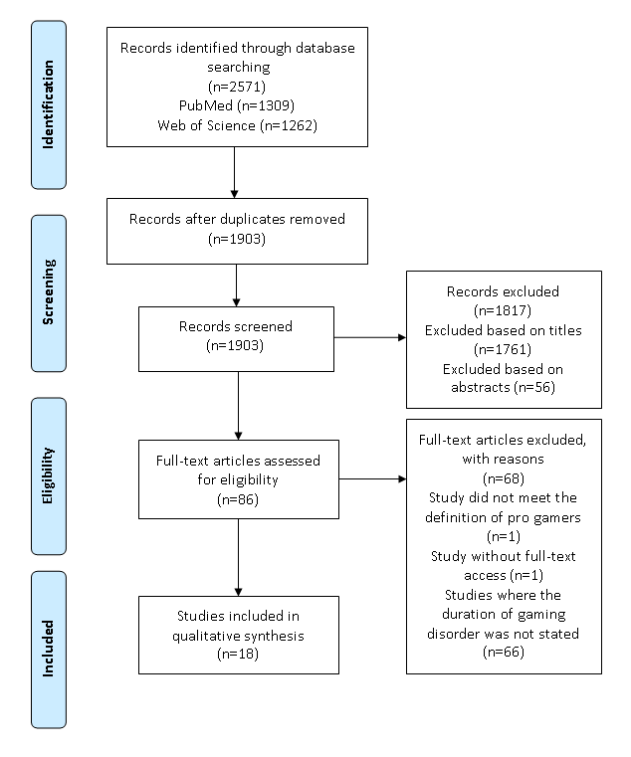
PRISMA (Preferred Reporting Items for Systematic Reviews and Meta-Analyses) flow diagram for the systematic literature review.

The searches of PubMed and Web of Science resulted in the identification of 2571 records. After the removal of duplicates, 1903 studies with unique titles were obtained for the screening of titles and abstracts. A total of 1761 studies were removed after the screening of titles, and another 56 studies were removed after the screening of abstracts. After excluding 1817 records, 86 full-text articles were comprehensively reviewed in order to assess their eligibility for inclusion in this review. After conducting assessments based on the inclusion criteria, 68 articles that did not meet the inclusion criteria or did not have full-text access were excluded (Figure 1). Thus, this review included 18 articles. The results of the extracted data from the selected articles are presented in three subsections: (1) information about the study, (2) alterations in brain regions in pro gamers, and (3) alterations in brain regions in individuals with GD.

### Information About the Study

#### Study Design

As seen in Table 1 [16,20,25,29-43], 16 studies had a cross-sectional design. One study [16] had a correlational design and one study [25] had a longitudinal design.

**Table 1:** Summary of 18 studies included in this review.

Authors; study design	Participant information	Brain-imaging technique	Alterations in brain regions associated with extensive video gaming
Hyun et al [16]; correlational	Pro gamers: N=23; all males; mean age 19.8 (SD 1.7) years	Magnetic resonance imaging (MRI)	Cortical thickness in the right superior frontal gyrus, the right superior parietal gyrus, and the right precentral gyrus
Tanaka et al [29]; cross-sectional	Pro gamers: N=17; all males; mean age 24.1 (SD 2.9) years Age- and educational level–matched control group: N=33; all males; weekly gaming duration was less than 2 hours	Structural MRI	Gray matter (GM) volume in the right posterior parietal cortex
Gong et al [30]; cross-sectional	Pro gamers: N=27; mean age 23.26 (SD 0.4) years Amateur players: N=30; mean age 22.3 (SD 0.38) years; did not habitually engage in video game playing, and video gaming experience was less than 1 year	MRI	Functional connectivity (FC) and GM volume in the insular subregions
Gong et al [31]; cross-sectional	Pro gamers: N=23; all males; mean age 23.3 (SD 4.3) years Amateur players: N=22; all males; mean age 22.3 (SD 3.46) years; video gaming experience was less than 1 year	Resting-state functional MRI (fMRI)	FC within and between the salience network (SN) and the central executive network (CEN)
Gong et al [20]; cross-sectional	Pro gamers: N=28; all males; mean age 24.6 (SD 1.4) years Amateurs players: N=30; all males; mean age 24.3 (SD 1.8) years	Diffusion tensor imaging (DTI)	White matter (WM) networks in the prefrontal network, the limbic system, and the sensorimotor network
Gong et al [25]; longitudinal	Pro gamers: N=20; all males; mean age 21.42 (SD 1.64) years Amateur players: N=20; all males; mean age 22.25 (SD 1.65) years; gaming experience was less than 1.5 years	Resting-state fMRI at the beginning and end of the study	Amplitude of low-frequency fluctuation (ALFF) in the brain regions of the default mode network (DMN), the CEN, and the SN
Han et al [32]; cross-sectional	Individuals with gaming disorder (GD): N=20; all males; mean age 20.9 (SD 2) years; mean duration of gaming disorder 4.9 (SD 0.9) years Pro gamers: N=17; all males; mean age 20.8 (SD 1.5) years Age- and educational level–matched healthy controls (HCs): N=18; all males; mean age 20.9 (SD 2.1) years	MRI	GM volume in cingulate gyrus, thalamus, and occipitotemporal areas
Ko et al [33]; cross-sectional	Participants with GD: N=15; mean age 24.67 (SD 3.11) years; mean education duration 15.47 (SD 1.56) years Remission subjects from GD: N=15; mean age 24.8 (SD 2.68) years; mean education duration 15.87 (SD 1.41) years HCs: N=15; mean age 24.47 (SD 2.83) years; mean education duration 16 (SD 1.13) years	Task-based fMRI (the presentation of neutral vs online game-related screenshots)	The bilateral dorsolateral prefrontal cortex (DLPFC), the precuneus, the left parahippocampus, the posterior cingulate, the right anterior cingulate, and the left superior parietal lobe
Yuan et al [34]; cross-sectional	Adolescents with GD: N=18; n=12 males; mean age 19.4 (SD 3.1) years Age- and gender-matched HCs: N=18; daily gaming duration was less than 2 hours	MRI	Cortical thickness in the left lateral orbitofrontal cortex, the insula cortex, lingual gyrus, the right postcentral gyrus, the entorhinal cortex, the inferior parietal cortex, the left precentral cortex, the middle temporal cortices, the precuneus, the middle frontal cortex, and the inferior temporal cortices
Yuan et al [35]; cross-sectional	Participants with GD: N=18; n=12 males; mean age 19.4 (SD 3.1) years Age- and gender-matched HCs: N=18; n=12 males; mean age 19.5 (SD 2.8) years; daily gaming duration was less than 2 hours	Resting-state fMRI	ALFF in brain regions, including major regions of the DMN
Ko et al [36]; cross-sectional	Male subjects with GD: N=26; mean age 24.58 (SD 3.23) years Male HCs: N=23; mean age 24.35 (SD 2.12) years	Task-state fMRI (go/no-go task)	Activation in the frontostriatal network
Chen et al [37]; cross-sectional	Individuals with GD: N=15; all males; mean age 24.67 (SD 3.12) years HCs: N=25; all males; mean age 24.47 (SD 2.83) years	Task-state fMRI (go/no-go task)	Activation in the right supplementary motor area (SMA) or pre-SMA
Ko et al [38]; cross-sectional	Male adults with GD: N=30; mean age 23.57 (SD 2.5) years Age- and educational level–matched HCs: N=30; mean age 24.23 (SD 2.47) years	Resting-state fMRI	GM density in bilateral amygdala FC of amygdala with the left DLPFC, the orbitofrontal lobe (OFL), and the contralateral insula
Cai et al [39]; cross-sectional	Individuals with GD: N=27; n=23 males; mean age 17.9 (SD 0.9) years Age-, gender-, and educational level–matched HCs: N=30; n=22 males; mean age 18.3 (SD 1.6) years	MRI	The volume in the striatum
Chen et al [40]; cross-sectional	Individuals with GD: N=28; all males; mean age 23.64 (SD 2.54) years Age- and educational level–matched HCs: N=28; all males; mean age 24.14 (SD 2.53) years	Resting-state fMRI	FC between the left insula and the left DLPFC and OFL, and between interhemispheric insula
Jin et al [41]; cross-sectional	College students with GD: N=25; n=16 males; mean age 19.12 (SD 1.05) years Age- and gender-matched HCs: N=21; n=14 males; mean age 18.76 (SD 1.81) years	Resting-state fMRI	GM volume in the prefrontal regions and the right SMA FC of prefrontal regions with temporal and occipital regions, and between several regions, including the bilateral caudate, the thalamus, the putamen, the insular cortex, and the right SMA
Yuan et al [42]; cross-sectional	Individuals with GD: N=43; n=32 males; age range 16-22 years; primary internet activity was to play League of Legends Age- and gender-matched HCs: N=44; n=34 males; age range 15-23 years	Resting-state fMRI	Volume in the striatum Resting-state FC within the dorsal and ventral striatum networks
Zhai et al [43]; cross-sectional	Subjects with GD: N=16; n=11 males; mean age 19.1 (SD 1.3) years; primary internet activity was to play League of Legends Age- and gender-matched HCs: N=16; n=11 males; mean age 18.4 (SD 1.9) years	DTI	The global and local efficiency of WM networks

#### Participants

Among the included 18 articles, six articles included pro gamers (Table 1). While pro gamers were compared with amateur video game players who had less gaming experience in five articles [20,25,29-31], one article [16] included pro gamers with varied gaming expertise. One article [32] included both pro gamers and individuals with GD. A total of 11 articles [33-43] included individuals with GD (Table 1). While one article [33] recruited a remission group in addition to individuals with GD and healthy controls, 10 articles recruited individuals with GD and healthy controls. Moreover, six articles [34,35,39,41-43] included female participants.

#### Brain-Imaging Techniques

Two studies used diffusion tensor imaging and six studies used magnetic resonance imaging to investigate the alterations in brain regions associated with extensive video game playing. Seven studies used resting-state functional magnetic resonance imaging (fMRI) and three studies used task-state fMRI. Tasks that were used in the studies included the presentation of gaming cues [33] and tasks to measure cognitive control [36,37] (Table 1).

### Alterations in Brain Regions in Pro Gamers

As seen in Table 1, pro gamers showed structural alterations in brain regions that were different from those in amateur players and individuals with GD. Pro gamers showed higher GM volume in the left cingulate gyrus [32], the right posterior parietal cortex (PPC) [29], and insula subregions, including the left long insular gyrus and central insular sulcus [30], compared to amateur players. The GM volume in the left cingulate gyrus was also higher in pro gamers than in individuals with GD [32]. Moreover, pro gamers showed decreased GM volume in some brain regions, including in the left middle occipital gyrus and the right inferior temporal gyrus, compared to healthy controls [32]. The GM volume in the left thalamus was lower in pro gamers than in individuals with GD [32]. Moreover, the pro gamers with longer career lengths were shown to have a thicker cortex in the right superior frontal gyrus, right superior parietal gyrus, and right precentral gyrus, and the pro gamers who won more in the competitions were shown to have a thicker PFC [16].

Pro gamers also showed functional alterations in brain regions. They showed increased amplitude of low-frequency fluctuation (ALFF) in brain regions of the default mode network (DMN), the central executive network (CEN), and the salience network (SN) compared to amateur players [25]. That is, pro gamers showed increased ALFF in the posterior cingulate cortex (PCC), the right angular gyrus, the right DLPFC, the anterior cingulated cortex (ACC), and the right anterior insula [25]. The WM network in the prefrontal regions, the limbic system, and the sensorimotor network was also more integrated in pro gamers compared to amateur players [20]. Moreover, pro gamers, compared to amateur players, showed more functionally connected networks, not only between anterior and posterior insula subregions [30] but also within and between the SN and CEN [31].

### Alterations in Brain Regions in Individuals With GD

Individuals with GD showed structural alterations compared to healthy controls and pro gamers (Table 1). Compared to both pro gamers and healthy controls, individuals with GD showed increased GM volume in the left thalamus [32]. Compared to healthy controls, individuals with GD showed increased volume in the striatum (ie, right caudate and right nucleus accumbens [NAc]) [39,42]; they also had a thicker cortex in the left precentral cortex, the middle temporal cortices, the precuneus, the middle frontal cortex, and the inferior temporal cortices [34]. Moreover, individuals with GD showed decreased GM volume in some brain regions compared to healthy controls. Individuals with GD were found to have decreased GM volume in the amygdala [38], the temporal-occipital cortex (ie, the right middle occipital gyrus and the left inferior occipital gyrus) [32], the prefrontal regions (ie, the bilateral DLPFC, the orbitofrontal cortex [OFC], and the ACC), and the right supplementary motor area (SMA) [41]. The cortical thickness in the left lateral OFC, the insula cortex, the lingual gyrus, the right postcentral gyrus, the entorhinal cortex, and the inferior parietal cortex was also found to be decreased in individuals with GD compared to healthy controls [34].

Individuals with GD showed functional changes in brain regions in the resting state compared to healthy controls. The short path length in individuals with GD was found to be increased [43]. Individuals with GD, compared to healthy controls, also showed increased ALFF in the left medial OFC, the left precuneus, the left SMA, the right parahippocampal gyrus, and the bilateral middle cingulate cortex [35]. Moreover, individuals with GD showed increases in the resting-state functional connectivity (FC), not only between the bilateral amygdala and the contralateral insula [38] but also between the bilateral insula [40].

Individuals with GD showed decreased resting-state activation or FC in some brain regions. The global and local efficiency of WM networks was found to be reduced in individuals with GD [43]. The FC between prefrontal regions (ie, ACC, OFC, and DLPFC) and temporal and occipital regions (ie, pallidum, thalamus, caudate, and putamen) also decreased in individuals with GD compared to healthy controls [41]. Moreover, individuals with GD, compared to healthy controls, showed both reduced FC in the dorsal and ventral striatum networks (ie, FC between the right caudate and the DLPFC, and FC between the right NAc and the OFC) [42] and decreased FC of the left insula with the left DLPFC and the orbitofrontal lobe (OFL) [40]. The FC of the bilateral amygdala with the left DLPFC and the FC of the right amygdala with the OFL were found to be decreased in individuals with GD [38].

Additionally, studies that used task-based fMRI showed that individuals with GD showed increased activation in the frontostriatal network (ie, bilateral OFL, ACC, left putamen, right DLPFC, and middle temporal lobe) [36] but decreased activation in the right SMA or pre-SMA [37] compared to healthy controls in the task that required inhibition. Furthermore, when gaming cues were presented, individuals with GD showed higher activation in the bilateral DLPFC, the precuneus, the left parahippocampus, the posterior cingulate, the right anterior cingulate, and the left superior parietal lobe compared to healthy controls [33]. Individuals with GD also showed higher activation in the right DLPFC, the left parahippocampus, and the left middle temporal gyrus in response to gaming cues compared to the remission group of participants [33].

## Discussion

### Overview

This review aimed to explore the association between extensive video game playing and changes in cognitive functions by focusing on pro gamers and individuals with GD. That is, this study systematically reviewed the brain-imaging studies that included pro gamers and/or individuals with GD. By following PRISMA guidelines, 18 studies were included in this review. Based on selected studies, it was found that pro gamers and individuals with GD showed different structural and functional alterations in brain regions, despite the comparable level of gaming engagement.

### Primary Results of the Studies Including Pro Gamers

Results showed both increased and decreased GM volume in brain regions of pro gamers. Pro gamers, compared to both healthy controls and individuals with GD, showed a thicker cortex in the left cingulate cortex, which is involved in the maintenance of attention and control over executive functioning [32]. Pro gamers also showed structural enhancement in brain regions that are involved in visual working memory, attention, and sensorimotor function (eg, the right PPC and insular subregions) compared to amateur video game players [29,30]. The increased GM volume in the right PPC was positively associated with better visual working memory performance in pro gamers [29], and the increased GM volume in insular subregions was suggested to contribute to functional integration within insular regions [30]. However, pro gamers showed decreased GM volume in occipitotemporal regions for visual processing (eg, the left middle occipital gyrus) compared to amateur players, and they showed decreased GM volume in the left thalamus, which is involved in reward processing, compared to individuals with GD [32]. These structural alterations in pro gamers suggested that pro gamers did not show impaired reward processing but showed enhanced cognitive function (eg, cognitive control and visual working memory), along with reduced cortical thickness in brain regions that are involved in the processing of visual stimuli. Moreover, since pro gamers—who reported longer video game experience or higher rates of winning in competitions—were found to show a thicker cortex in frontal regions for cognitive flexibility (eg, the right superior frontal gyrus) [16], it was suggested that gaming expertise seemed to modulate the association between extensive video game experience and cognitive enhancement in pro gamers.

Results also showed functional enhancement in brain regions of pro gamers. Pro gamers showed more functional integration between anterior and posterior insular subregions [30] and within and between the SN and the CEN [31]. That is, the attention and sensorimotor functions were more coordinated in pro gamers [30], and they showed improvement in the ability to process information [31]. The plasticity of WM networks in brain regions for sensorimotor function and cognitive control (eg, the sensorimotor network and the prefrontal network) was also more enhanced in pro gamers compared to amateur players [20]. Pro gamers were found to integrate information more efficiently by showing nodal and global enhancement in WM networks [20]. Moreover, it was found that activation in the DMN (eg, PCC), the CEN (eg, the right DLPFC), and the SN (eg, the ACC), which was higher in pro gamers than in amateur players at the beginning of the study, decreased after the pro gamers were asked not to play video games for 1 year [25]. The results of that longitudinal study suggested that extensive video game playing seemed to enhance the development of brain regions [25]. Consistent with structural alterations in pro gamers, when compared to amateur players, pro gamers showed functional enhancements within and between the brain regions that are involved in attention, visual processing, sensorimotor function, and cognitive control.

### Primary Results of the Studies Including Individuals With GD

Individuals with GD were found to show structural alterations in frontostriatal regions. While the cortical thickness of the brain regions that were associated with executive function and decision making (eg, the DLPFC, the OFC, and the amygdala) decreased in those with GD [34,38,41], the volumes of the brain regions for reward processing (eg, the striatum) increased in individuals with GD as compared to healthy controls [39,42]. Individuals with GD also showed increased volumes in the brain region that is involved in the expectation of rewards (ie, the left thalamus) compared to pro gamers [32]. The structural alterations in brain regions of individuals with GD suggested that they showed impairment in executive functioning and higher levels of craving to play video games [34]. In particular, the increased volume in the striatum was found to be associated with impairment of cognitive control [39]. However, individuals with GD showed increased volume in the middle temporal cortex, which is involved in the acquisition of skills, compared to healthy controls [34]. That is, despite impaired cognitive control in individuals with GD, their higher level of video game playing experience, compared to that of healthy controls, was associated with improved skills.

Individuals with GD were found to show not only structural alterations but also functional alterations compared to healthy controls. Individuals with GD showed reduced levels of integration within WM networks [43]. The increased short path length [43] and increased resting-state FC within and between the bilateral insula and the amygdala [38,40] in individuals with GD were found to be associated with a higher level of impulsivity. An increased resting-state activation in certain brain regions (eg, the left medial OFC) [35] was also associated with impairment of cognitive control in individuals with GD. Moreover, the CEN (eg, DLPFC) and the reward circuits (eg, amygdala) in individuals with GD, compared to healthy controls, were found to be less functionally integrated in the resting state [38,40]. That is, the FC within the frontostriatal networks, which are involved in the processing of motivation and cognitive control, was found to decrease in individuals with GD during the resting state [41,42]. It was suggested that they showed both higher levels of impulsivity and impaired cognitive control.

Consistent with the resting-state functional alterations in individuals with GD, the task-state fMRI studies showed impairment of cognitive control in individuals with GD. When the execution of cognitive control was required, individuals with GD showed increased activation in the frontostriatal networks, unlike healthy controls who showed increased activation only in the DLPFC [36]. That is, not only frontal regions but also striatal regions were activated in individuals with GD for response inhibition. However, activation in the brain region that is involved in executing proper behavior (eg, pre-SMA) decreased in individuals with GD in the cognitive control task [37]. Moreover, when the gaming cue was presented, individuals with GD showed increased activation in brain regions that are involved in the processing of affective or salient stimuli and craving (eg, the bilateral DLPFC, the posterior cingulate, and the anterior cingulate) compared to healthy controls [33]. The remission group, who showed lower levels of craving video game playing than individuals with GD, also showed higher activation in the brain region for visual attention (ie, the superior parietal lobe), though the difference in activation between the remission group and healthy controls was not significant [33].

Furthermore, there was a difference in structural and functional alterations in brain regions among individuals with GD. Individuals who reported more severe levels of GD showed an increased volume in the NAc, which is involved in the processing of rewards [39]. Individuals who reported having GD for a longer duration showed not only an increased volume in the left precentral gyrus and precuneus but also a decreased volume in the lingual gyrus [34]. They also showed more abnormal resting-state activation in the left medial OFC and the left precuneus [35]. Moreover, the level of self-control in individuals with GD was negatively associated with activation of the bilateral caudate nucleus [36]. These findings suggest that it was plausible that the severity or duration of GD and impaired self-control mediated the association between extensive video game playing and changes in cognitive function.

Taken together, pro gamers and individuals with GD showed different structural and functional alterations in brain regions despite comparable extensive engagement in video game playing. Pro gamers showed enhancement in cognitive function (eg, attention and visuomotor function) and better cognitive control. Unlike pro gamers, individuals with GD showed impairment in cognitive control and higher levels of craving video game playing. They also showed improvement in the acquisition of skills. Moreover, factors that seemed to modulate the association of extensive video game playing with changes in cognitive function (ie, gaming expertise, duration or severity of GD, and level of self-control) were identified. That is, although individuals with GD showed impaired executive functioning, extensive video game playing was associated with enhancement in cognitive function in not only pro gamers but also in those with GD.

### Limitations

There were three limitations in this review. The first limitation was the limited number of studies. Only a few studies were identified that included pro gamers. Most studies that included individuals with GD did not consider or state the duration of GD despite its importance. According to the WHO’s announcement, the diagnosis of GD should be based on the report of symptoms for more than 12 months [7]; only a subgroup of individuals were found to show persistent symptoms of GD over 12 months [44]. That is, more studies should be conducted that include pro gamers and individuals who had reported GD for more than 1 year. The second limitation was the design of the studies. Although one study had a longitudinal design, most included studies had cross-sectional designs. More brain-imaging studies with longitudinal designs should be conducted, as these would be helpful in tracking alterations in brain regions and in understanding causality in the association between extensive video game playing and changes in cognitive function. The last limitation was the comparison group. Most included studies recruited participants who did not habitually play video games as the comparison group. There was a group of highly engaged video game players who were not pro gamers and did not show any symptoms of GD [45]; therefore, comparing alterations in brain regions between individuals with GD and highly engaged video game players or pro gamers would be helpful to deepen the understanding of the effect of video game playing on structural and functional alterations in brain regions and to identify the mediating factors of their association. Further studies should be conducted while considering these limitations.

### Conclusions

Pro gamers and individuals with GD showed differences in structural and functional alterations in certain brain regions. While pro gamers showed enhancement in cognitive functions (eg, cognitive control), individuals with GD showed impaired cognitive control despite the acquisition of better skills compared to non–video game players. Mediating factors (eg, the duration of GD) were found to be associated with different alterations of brain regions in pro gamers and individuals with GD. That is, it was suggested that various factors seemed to modulate the association of extensive video game playing with changes in cognitive function. However, a limited number of brain-imaging studies included pro gamers and/or individuals who reported symptoms of GD for more than 1 year. Thus, more studies that include pro gamers and/or individuals with GD, as well as more diverse comparison groups, and ones that longitudinally track alterations in brain regions should be conducted in the future.

## Abbreviations

ACC: anterior cingulate cortex
AI: artificial intelligence
ALFF: amplitude of low-frequency fluctuation
CEN: central executive network
DLPFC: dorsolateral prefrontal cortex
DMN: default mode network
FC: functional connectivity
fMRI: functional magnetic resonance imaging
GD: gaming disorder
GM: gray matter
ICD-11: International Classification of Diseases, 11th Revision
NAc: nucleus accumbens
OFC: orbitofrontal cortex
OFL: orbitofrontal lobe
PCC: posterior cingulate cortex
PFC: prefrontal cortex
PHR: personal health record
PPC: posterior parietal cortex
PRISMA: Preferred Reporting Items for Systematic Reviews and Meta-Analyses
SMA: supplementary motor area
SN: salience network
WHO: World Health Organization
WM: white matter

## References

[1] Lee D, Schoenstedt LJ (2011). Comparison of eSports and traditional sports consumption motives. J Res.

[2] (2005). S Korean dies after gaming session. BBC News.

[3] (2011). Chinese online gamer dies after three-day session. BBC News.

[4] (2019). My gaming addiction stops me from having relationships. BBC News.

[5] Good OS (2019). 'Gaming disorder' officially on World Health Organization's list of diseases. Polygon.

[6] Rettner R (2019). Video game addiction becomes official mental disorder in controversial decision by WHO. Live Science.

[7] (2018). Addictive behaviors: Gaming disorder. World Health Organization.

[8] (2019). Video game addiction is a mental health disorder, World Health Organization says. NBC News.

[9] Kim HJ (2019). Psychologists express concern over registration of game disorder disease code. ZDNet Korea.

[10] Park G (2019). 'Game addiction' altering the structure of the brain, the importance of early treatment. KBS News.

[11] Pan N, Yang Y, Du X, Qi X, Du G, Zhang Y, Li X, Zhang Q (2018). Brain structures associated with internet addiction tendency in adolescent online game players. Front Psychiatry.

[12] Kühn S, Lorenz R, Banaschewski T, Barker GJ, Büchel C, Conrod PJ, Flor H, Garavan H, Ittermann B, Loth E, Mann K, Nees F, Artiges E, Paus T, Rietschel M, Smolka MN, Ströhle A, Walaszek B, Schumann G, Heinz A, Gallinat J, IMAGEN Consortium (2014). Positive association of video game playing with left frontal cortical thickness in adolescents. PLoS One.

[13] Kühn S, Gallinat J (2014). Amount of lifetime video gaming is positively associated with entorhinal, hippocampal and occipital volume. Mol Psychiatry.

[14] Zhang Y, Du G, Yang Y, Qin W, Li X, Zhang Q (2015). Higher integrity of the motor and visual pathways in long-term video game players. Front Hum Neurosci.

[15] Richlan F, Schubert J, Mayer R, Hutzler F, Kronbichler M (2018). Action video gaming and the brain: fMRI effects without behavioral effects in visual and verbal cognitive tasks. Brain Behav.

[16] Hyun GJ, Shin YW, Kim B, Cheong JH, Jin SN, Han DH (2013). Increased cortical thickness in professional on-line gamers. Psychiatry Investig.

[17] Lee YH, Jung MG, Jang MJ, Lee JJ, Nam GD, Han JO, Lim TJ, Yun DJ, Yoo HG, Kim SP (2018). The 2018 Survey on the Korean E-sports Industry.

[18] Chapman L (2018). Salaries of pro gamers. CHRON.

[19] Thompson JJ, Blair MR, Henrey AJ (2014). Over the hill at 24: Persistent age-related cognitive-motor decline in reaction times in an ecologically valid video game task begins in early adulthood. PLoS One.

[20] Gong D, Ma W, Gong J, He H, Dong L, Zhang D, Li J, Luo C, Yao D (2017). Action video game experience related to altered large-scale white matter networks. Neural Plast.

[21] Leeman RF, Potenza MN (2013). A targeted review of the neurobiology and genetics of behavioural addictions: An emerging area of research. Can J Psychiatry.

[22] Wei L, Zhang S, Turel O, Bechara A, He Q (2017). A tripartite neurocognitive model of internet gaming disorder. Front Psychiatry.

[23] Moher D, Liberati A, Tetzlaff J, Altman DG (2009). Preferred reporting items for systematic reviews and meta-analyses: The PRISMA statement. PLoS Med.

[24] Wang Y, Yin Y, Sun YW, Zhou Y, Chen X, Ding WN, Wang W, Li W, Xu JR, Du YS (2015). Decreased prefrontal lobe interhemispheric functional connectivity in adolescents with internet gaming disorder: A primary study using resting-state FMRI. PLoS One.

[25] Gong D, Yao Y, Gan X, Peng Y, Ma W, Yao D (2019). A reduction in video gaming time produced a decrease in brain activity. Front Hum Neurosci.

[26] Bremer J (2005). The internet and children: Advantages and disadvantages. Child Adolesc Psychiatr Clin N Am.

[27] Steinberg L (2005). Cognitive and affective development in adolescence. Trends Cogn Sci.

[28] Cho YU, Lee D, Lee JE, Kim KH, Lee DY, Jung YC (2017). Exploratory metabolomics of biomarker identification for the internet gaming disorder in young Korean males. J Chromatogr B Analyt Technol Biomed Life Sci.

[29] Tanaka S, Ikeda H, Kasahara K, Kato R, Tsubomi H, Sugawara SK, Mori M, Hanakawa T, Sadato N, Honda M, Watanabe K (2013). Larger right posterior parietal volume in action video game experts: A behavioral and voxel-based morphometry (VBM) study. PLoS One.

[30] Gong D, He H, Liu D, Ma W, Dong L, Luo C, Yao D (2015). Enhanced functional connectivity and increased gray matter volume of insula related to action video game playing. Sci Rep.

[31] Gong D, He H, Ma W, Liu D, Huang M, Dong L, Gong J, Li J, Luo C, Yao D (2016). Functional integration between salience and central executive networks: A role for action video game experience. Neural Plast.

[32] Han DH, Lyoo IK, Renshaw PF (2012). Differential regional gray matter volumes in patients with on-line game addiction and professional gamers. J Psychiatr Res.

[33] Ko CH, Liu GC, Yen JY, Chen CY, Yen CF, Chen CS (2013). Brain correlates of craving for online gaming under cue exposure in subjects with internet gaming addiction and in remitted subjects. Addict Biol.

[34] Yuan K, Cheng P, Dong T, Bi Y, Xing L, Yu D, Zhao L, Dong M, von Deneen KM, Liu Y, Qin W, Tian J (2013). Cortical thickness abnormalities in late adolescence with online gaming addiction. PLoS One.

[35] Yuan K, Jin C, Cheng P, Yang X, Dong T, Bi Y, Xing L, von Deneen KM, Yu D, Liu J, Liang J, Cheng T, Qin W, Tian J (2013). Amplitude of low frequency fluctuation abnormalities in adolescents with online gaming addiction. PLoS One.

[36] Ko CH, Hsieh TJ, Chen CY, Yen CF, Chen CS, Yen JY, Wang PW, Liu GC (2014). Altered brain activation during response inhibition and error processing in subjects with internet gaming disorder: A functional magnetic imaging study. Eur Arch Psychiatry Clin Neurosci.

[37] Chen CY, Huang MF, Yen JY, Chen CS, Liu GC, Yen CF, Ko CH (2015). Brain correlates of response inhibition in internet gaming disorder. Psychiatry Clin Neurosci.

[38] Ko CH, Hsieh TJ, Wang PW, Lin WC, Yen CF, Chen CS, Yen JY (2015). Altered gray matter density and disrupted functional connectivity of the amygdala in adults with internet gaming disorder. Prog Neuropsychopharmacol Biol Psychiatry.

[39] Cai C, Yuan K, Yin J, Feng D, Bi Y, Li Y, Yu D, Jin C, Qin W, Tian J (2016). Striatum morphometry is associated with cognitive control deficits and symptom severity in internet gaming disorder. Brain Imaging Behav.

[40] Chen CY, Yen JY, Wang PW, Liu GC, Yen CF, Ko CH (2016). Altered functional connectivity of the insula and nucleus accumbens in internet gaming disorder: A resting state fMRI study. Eur Addict Res.

[41] Jin C, Zhang T, Cai C, Bi Y, Li Y, Yu D, Zhang M, Yuan K (2016). Abnormal prefrontal cortex resting state functional connectivity and severity of internet gaming disorder. Brain Imaging Behav.

[42] Yuan K, Yu D, Cai C, Feng D, Li Y, Bi Y, Liu J, Zhang Y, Jin C, Li L, Qin W, Tian J (2017). Frontostriatal circuits, resting state functional connectivity and cognitive control in internet gaming disorder. Addict Biol.

[43] Zhai J, Luo L, Qiu L, Kang Y, Liu B, Yu D, Lu X, Yuan K (2017). The topological organization of white matter network in internet gaming disorder individuals. Brain Imaging Behav.

[44] Wartberg L, Kriston L, Zieglmeier M, Lincoln T, Kammerl R (2019). A longitudinal study on psychosocial causes and consequences of internet gaming disorder in adolescence. Psychol Med.

[45] King DL, Delfabbro PH (2016). The cognitive psychopathology of internet gaming disorder in adolescence. J Abnorm Child Psychol.

